# Sexualized drug injection among men who have sex with men in Madrid and Barcelona as the first episode of drug injecting

**DOI:** 10.1186/s12954-021-00531-2

**Published:** 2021-08-06

**Authors:** Juan-Miguel Guerras, Patricia García de Olalla, María José Belza, Luis de la Fuente, David Palma, Jorge del Romero, Jorge-Néstor García-Pérez, Juan Hoyos, the Methysos Project Group, Marta Donat, María del Carmen Burgos, César Pérez Romero, José Antonio San Juan Bueno, Francisca Román Urrestarazu, Jesus E Ospina, Miguel Alarcón Gutiérrez, Oskar Ayerdi, Carmen Rodríguez, Sonsoles del Corral Del Campo, Natividad Jerez Zamora, Marta Ruiz Fernández, Montserrat González Polo, María Jesús Barbera Gracia, Luis López Pérez, Claudia Broto Cortes, Julio Morais Martin

**Affiliations:** 1 Centro Nacional de Epidemiología, Instituto de Salud Carlos III, Madrid, Spain; 2 CIBER Epidemiologia Y Salud Pública (CIBERESP), Madrid, Spain; 3 Servicio de Epidemiología, Agència de Salut Pública de Barcelona, Barcelona, Spain; 4 Escuela Nacional de Sanidad, Instituto de Salud Carlos III, Monforte de Lemos 5, 28029 Madrid, Spain; 5 Centro Sanitario Sandoval, Instituto de Investigación Sanitaria San Carlos, Hospital Clínico San Carlos, Madrid, Spain; 6 Unidad de ITS de Vall D’Hebron-Drassanes, Hospital Vall D’Hebron, Barcelona, Spain; 7 Departamento de Salud Pública Y Materno-Infantil, Universidad Complutense de Madrid, Madrid, Spain

**Keywords:** Drug injection, MSM, Chemsex, Slamming

## Abstract

**Background:**

We estimate the prevalence of drug injection, the variables associated with having ever injected and the proportion of ever injectors whose first drug injection was for having sex; we describe the first drug injection episode, analyze the drugs most frequently injected and estimate the prevalence of risky injecting behaviors.

**Methods:**

The participants were 3387 MSM without a previous HIV diagnosis attending four HIV/STI diagnosis services in Madrid and Barcelona. Lifetime prevalence and prevalence ratios (PRs) by different factors were calculated using Poisson regression models with robust variance. We compared the characteristics of first drug injection episode, lifetime injection and risky injecting behaviors of those whose first injection was for sex (FIS) with those whose was not (non-FIS).

**Results:**

Lifetime prevalence of injection was 2.1% (CI 1.7–2.7). In the multivariate analysis, it was strongly associated with having been penetrated by more than five men in the last 12 months (aPR = 10.4; CI 2.5–43.4) and having met most of their partners at private parties (aPR = 7.5; CI 4.5–12.3), and less strongly with other factors. Of those who had ever injected drugs, 81.9% injected for sex the first time they injected drugs (FIS). At first injection, FIS participants had a mean age of 31 years, 62.7% used mephedrone and 32.2% methamphetamine on that occasion. Of this FIS group 39.0% had ever shared drugs or equipment and 82.6% had always shared for sex. Some 30.8% of non-FIS reported having also injected drugs for sex later on.

**Conclusions:**

Only two out of a hundred had ever injected, most to have sex and with frequent drug or injecting equipment sharing. Injecting for sex is the most common first episode of drug injection and is the most efficient risky behavior for the transmission of HIV, hepatitis B or C and other blood-borne infections. MSM participating in private parties should be considered a priority group for prevention policies.

**Supplementary Information:**

The online version contains supplementary material available at 10.1186/s12954-021-00531-2.

## Introduction

During the last two decades of the 20st century, Spain was arguably the developed country most heavily affected by two intertwined epidemics: first, a drug injection epidemic (mostly heroin) [1]; immediately followed by an HIV epidemic [2, 3]. As a consequence, Spain was for years one of the countries with the highest rates of AIDS cases linked to intravenous drug use [4]. The incidences of these two epidemics peaked before most drug users were even aware of the HIV-infection risk. Moreover, also before users became generally aware, a transition in heroin administration route had begun in Southwest Spain which gradually spread to the northeast; injection was replaced by “chasing the dragon” (inhaling the vapors which result when the drug is heated) as both the initial and usual administration route [5]. Madrid and Barcelona experienced very different patterns in the development of injection practices. Madrid began the transition from injection to chasing the dragon before Barcelona and the latter maintained a higher level of administration by injection [6]. HIV-prevention programs in the second part of the eighties and especially the development of harm reduction policies during the nineties [3] were decisive for a radical decrease in the prevalence and especially the incidence of injection. These policies also led to a reduction in new HIV diagnoses linked to this risky behavior: only 2.6% in 2019 [7].

In the first two decades of the current century, there has not been any consistent evidence of an increase in drug-injection incidence in Spain. However, in the last decade several studies—either conducted only in Spain, or as part of European studies—have shown that Spanish men who have sex with other men (MSM) are adopting new patterns of sexualized drug-use characterized by the use of specific substances (mainly methamphetamine, mephedrone, GHB/GBL or ketamine) generally labeled “chemsex drugs” (ChSD) [8–11]. Although the characteristics of the participants in the studies are very heterogeneous, practically all of those that addressed the topic showed some evidence of associations between chemsex behavior and higher prevalence of HIV, HCV and other sexually transmitted infection (STI) [8, 11–14]. Administration routes for these substances are generally snorting or ingesting, but they are also sometimes injected, a practice known as “slamming.” This administration route is motivated by similar reasons to chemsex, but also for some specific reasons [14]. Available epidemiological analysis of slamming is limited, with many studies being qualitative or having a fairly limited sample size [14–18]. Moreover, nearly all the research overall is focused on the association with sexual risk practices, and, as far as we know, no analysis of initiation circumstances has yet been undertaken. Thus, the proportion of MSM who have ever injected drugs who report that their first episode of drug injecting was for having sex (slamming) remains unknown. Likewise, the percentage who report having injected for the first time for purposes other than having sex is also unknown. Nor has it been studied whether and to what extent the process of preparing and injecting the substances implies sharing of needles, syringes or other devices, although it has been suggested that at least certain subpopulations often share syringes [19]. It is well known that this risky behavior is much more efficient than sexual practices at transmitting HIV, HBV and HCV [20], and, for pharmacokinetic reasons, it is one of the strongest correlates for overdose [21, 22] and the development of dependence [23].

This original is focused on MSM without a previous positive HIV diagnosis, while it is still possible to prevent this infection; HCV, whose prevalence is extremely low in HIV negative MSM [24] may also be prevented. It has been carried out in Madrid and Barcelona: the two most populated cities in Spain which also have high prevalences of MSM, because they attract MSM both from other parts of Spain and abroad.

We estimate the prevalence of drug injection in different periods and the variables associated with having ever injected; we also estimate the proportion of ever injectors whose first drug injection in their lives was for having sex; we describe this first injection episode, analyze the drugs that have ever and most frequently been injected, and estimate the prevalence of risky injecting behaviors.

## Methods

### Design, recruitment, data collection instruments and variables

This survey was the first design included in the Methysos Project, which is devoted to analyzing the prevalence and characteristics of drug use (including sexualized drug use) in MSM in Spain. A cross-sectional survey was carried out in four facilities: the two most important sexually transmitted infection clinics in Spain—Sandoval, in Madrid and Drassanes, in Barcelona—and two community programs for rapid HIV-testing: Pink Peace Program, in Madrid, and Agencia de Salut Pública, in Barcelona. The STI clinics basically provide on-demand services and perform classical testing for all STIs, whereas the community programs also carry out various different kinds of active recruitment, including via ads and profiles on dating apps for MSM, and only offer rapid testing for HIV, syphilis and, sometimes, for HCV.

The recruitment began in May 2018 and finished in December 2020. The study was restricted to MSM without a previous HIV diagnosis, because they make up the vast majority of attendees and the priority group for prevention of both HIV and HCV. Therefore, only those MSM without a previous HIV test in their life or those whose last HIV test had been negative accessing these facilities were offered to participate. If they accepted, they answered a self-administered online questionnaire without personal identifiers on a tablet. The questionnaire had different sections: sociodemographics, history of HIV testing, sexual risk behaviors and both recreational and sexualized drug use. It included a short set of questions related with drug injection for recreational use or in a sexual context: “Have you ever injected any drug?”, characteristics of first injection, some characteristics of having ever injected, and of “sharing” material to prepare drugs or sharing injection equipment. The term “sharing” includes several behaviors, because in the questionnaire it was defined as “having injected with a syringe previously used by another person; or, having taken the dissolved drug from a syringe previously used by another person; or, from the recipient in which another person has previously inserted their syringe.” The study was approved by the Research Ethics Committee of the Instituto de Salud Carlos III (CEI PI 44_2018_subproyecto1-v2 and CEI PI 44_2018_subproyecto2).

### Statistical analysis

Nearly all of the variables were collected in a more disaggregated form than presented here, since some of the original categories have been grouped together based on their frequencies and the rationale for the analysis. See Additional file 1 for more information on how original variables and categories from the questionnaire were managed to obtain the final variables and categories used in the analysis. Most of the final variables were based only on one original variable. Characteristics (n and percentages) of the participants were described: sociodemographics, sexual risk behaviors, history of HIV testing and STI diagnosis. Then, we calculated the prevalence of drug injection for different periods, and the prevalence of having ever injected stratified by different variables. Comparisons of independent variables were assessed using Pearson’s *χ*^2^ and Fisher’s exact tests. For the analysis of correlates of ever having injected, Poisson regression models with robust variance were used [25, 26]. Both crude and adjusted Prevalence Ratios (cPRs and aPRs) and 95% Confidence Intervals (95%CI) were calculated. Variables with a significance level of < 0.25 in the bivariate analysis were introduced into the multivariate model—after collapsing the number of categories for many of these variables, due to the limited number of participants who had ever injected. We used the Akaike Information Criteria to perform model comparisons in order to select the final model. Finally, we stratified the participants into two groups: those whose first injection was for sex (FIS) and those whose was not (non-FIS), and we made a comparison of the different characteristics of the first injection episode, drugs injected for all purposes and specifically for sexual purposes, frequency of injection and “sharing” of drugs or injection equipment between the two groups.

## Results

As shown in Table 1, the study included 3387 participants: 2356 in Madrid (1224 in the STI center and 1132 in the community center) and 1031 in Barcelona (418 in the STI center and 613 in the community center). Concerning sociodemographics: 74.0% of the participants were under 40 years of age, 37.9% were born abroad, 75.6% lived in the municipalities of Madrid or Barcelona, 59.8% had university-level studies, 59.8% had a comfortable economic situation and 40.4% had lived alone during the last 12 months.

**Table 1:** Sample characteristics and bivariate analysis of factors associated with having ever injected drugs among MSM* in Madrid and Barcelona

	Sample characteristics (*N* = 3387)		Prevalence of having ever injected drugs (2.1%)	Crude prevalence ratio	(95% CI**)
	N	%	%		
Recruitment					
*City of testing*					
Barcelona	1031	30.4	1.6	1.0	
Madrid	2356	69.6	2.4	1.5	0.9–2.7
*Kind of testing program*					
STI diagnostic center	1642	48.5	1.7	1.0	
Comunity program	1745	51.5	2.5	1.5	0.9–2.4
Sociodemographics					
*Age (years)*					
≤ 24	559	16.5	2.0	1.3	0.6–3.0
25–39	1949	57.5	2.5	1.6	0.9–3.1
≥ 40	879	26.0	1.5	1.0	
*Country of birth*					
Spain	2105	62.1	1.8	1.0	
Latin-America	927	27.4	3.0	1.7	1.0–2.7
Others	355	10.5	1.7	0.9	0.4–2.1
*Years residing in Spain (for those born outside of Spain)*					
≤ 1	332	29.2	2.4		
2–4	281	24.7	2.9		
≥ 5	526	46.2	2.9		
*Size of city of residence (last 12 months)*					
≤ 100.000	389	11.5	1.5	1.0	
100.000–1 million	434	12.9	1.8	1.2	0.4–3.4
> 1 million	2547	75.6	2.3	1.5	0.6–3.4
*Level of education*					
Up to upper secondary	229	6.8	3.1	1.8	0.8–4.0
Post secondary	1129	33.5	2.7	1.5	0.9–2.5
University	2016	59.8	1.7	1.0	
Employment status (last 12 months)***					
Employed	1737	74.1	2.3	1.0	
Unemployed	183	7.8	6.0	2.7	1.4–5.2
Others	424	18.1	1.4	0.6	0.3–1.5
*Economic situation (last 12 months)*					
Comfortable/it is OK	2022	59.8	2.2	1.0	
Tight	1070	31.7	2.1	0.9	0.6–1.6
Dificult/very difficult	287	8.5	2.1	1.0	0.4–2.3
Cohabitation (last 12 months)***					
Alone	947	40.4	2.9	1.0	
With some people	1400	59.6	2.1	0.7	0.4–1.2
Sexual and risk behavior					
*Gender of sex partners (ever)*					
Only men	2119	62.6	1.5	1.0	
Men and women	1268	37.4	3.2	2.1	1.3–3.3
*Age at first sexual intercourse with another men (years)*					
< 16	596	17.6	3.5	7.3	1.7–31.0
16–20	1826	54.0	2.2	4.5	1.1–18.7
21–24	549	16.2	1.6	3.4	0.7–15.6
≥ 25	412	12.2	0.5	1.0	
*Lives sex-life with men…*					
Not Openly	1297	38.5	1.4	1.0	
Openly	2075	61.5	2.6	1.9	1.1–3.2
*Place where the largest number of partners were met*					
Dicos/clubs/bars	509	15.6	1.4		
Saunas	320	9.8	1.9		
Apps/websites	2093	64.2	1.8		
Cruising places	103	3.2	1.9		
Private parties	112	3.4	17.9		
Others/no search	122	3.7	0.0		
*Place where the largest number of partners were met*					
Others	3147	96.6	1.7	1.0	
Private parties	112	3.4	17.9	10.8	6.5–18.1
*Number of men penetrated by (ever)*					
None	178	5.3	0.6	1.0	
1–49	2345	69.2	1.3	2.4	0.3–17.2
≥ 50	864	25.5	4.6	8.2	1.1–59.9
*Number of men penetrated by (last 12 months)*					
None	691	20.4	0.3	1.0	
≤ 5	1569	46.4	1.5	5.1	1.2–21.5
> 5	1123	33.2	4.2	14.5	3.5–59.5
*Been paid for sex*					
Never	2660	78.6	1.5	1.0	
> 12 months ago	321	9.5	2.8	1.8	0.9–3.7
Last 12 months	405	12.0	5.4	3.5	2.1–5.9
*Paid for sex*					
Never	2805	82.9	2.0	1.0	
> 12 months ago	290	8.6	2.1	1.0	0.4–2.4
Last 12 months	290	8.6	3.4	1.7	0.9–3.4
*Ever injected steroids*					
No	3177	93.8	1.8	1.0	
Yes	210	6.2	6.7	3.7	2.0–6.5
History of HIV and other STI testing					
*Time since last HIV test*					
Never tested before	218	6.4	0.5	1.0	
< 6 months	1661	49.1	2.2	4.9	0.7–35.4
> 6 months	1502	44.4	2.3	4.9	0.7–36.0
*HIV new diagnosis in this consultation*					
No	3253	97.5	2.0	1.0	
Yes	82	2.5	3.7	1.9	0.6–5.9
*STI diagnosis (ever)*					
No	955	28.5	0.8	1.0	
Yes	2399	71.5	2.7	3.2	1.5–6.6

*MSM: men who have sex with men

**95% CI: 95% confidence interval

***These question were not included in Barcelona

With respect to sexual and risk behavior: 62.6% had only ever had sex with men, 17.6% had had their first sexual relationship with a man before 16 years of age, 61.5% lived their sexual life with men openly, 64.2% had met most of their partners through websites or dating apps; 5.3% had never been penetrated and 25.5% had been penetrated by more than 50 men in their lives; 21.5% had been paid for sex; 17.2% had paid for sex; and 6.2% had injected steroids. Concerning HIV and STI testing: 49.1% had been tested for HIV in the last 6 months and only 6.4% had never been tested before; 2.5% were diagnosed with an HIV infection in that consultation, and 71.5% had been diagnosed with an STI at some point.

Lifetime injection prevalence for any drug in the global sample was 2.1% (CI 1.7–2.7), which was higher, but not significantly, in Madrid (2.4%; CI 1.9–3.1) than Barcelona (1.6; CI 0.9–2.6) and in the community programs (2.5%, CI 1.9–3.4) than in the STI centers (1.7%; CI 1.2–2.5). In the crude analysis (Table 1), the prevalence of lifetime injection was significantly associated (when the 95% CI of the cPR did not include the null value, 1) with being born in Latin America (cPR = 1.7; CI 1.0–2.7), being unemployed (cPR = 2.7; CI 1.4–5.2), ever having had sex with women (cPR = 2.1; CI 1.3–3.3), having had their first sexual intercourse with a man before age 20 (cPR = 1.8; CI 1.0–3.1), living their sexual life openly (cPR = 1.9; CI 1.1–3.2), having found most of their sexual partners in private parties (cPR = 10.8; CI 6.5–18.1), having been penetrated by more than fifty men in their lives, (cPR = 8.2; CI 1.1–59-9), having been penetrated by more than five men in the last 12 months (cPR = 14.5; CI 3.5–59.5), having been paid for sex in the last 12 months (cPR = 3.5; CI 2.1–5.9), ever having injected steroids (cPR = 3.7; CI 2.0 6.5), and having been diagnosed with an STI (cPR = 3.2; CI 1.5–6.6).

In the multivariate analysis (Table 2) higher prevalence of injection remained independently and strongly associated with two variables: Having been penetrated by more than five men in the last 12 months (aPR = 10.4; CI 2.5–43.4), and having found most of their sexual partners in private parties (aPR = 7.5; CI 4.5–12.3). It also remained associated with another four variables, but with much lower aPRs, around two: ever having injected steroids, ever having been diagnosed with an STI, ever having been paid for sex, and having also had sex with women.

**Table 2:** Multivariate regression analysis of factors associated with having ever injected drugs among MSM* in Madrid and Barcelona (*N* = 3387)

	aPR**	(95% CI***)
*Number of men who had penetrated him (last 12 months)*		
None	1.0	
≤ 5	5.1	1.3–21.7
> 5	10.4	2.5–43.4
*Place where the largest number of partners were met*		
Others	1.0	
Private parties	7.5	4.5–12.3
*Ever injected steroids*		
No	1.0	
Yes	2.3	1.3–4.2
*STI diagnosis (ever)*		
No	1.0	
Yes	2.2	1.1–4.6
*Been paid for sex*		
Never	1.0	
> 12 months ago	1.3	0.7–2.7
Last 12 months	2.1	1.3–3.5
*Gender of sex partners (ever)*		
Only men	1.0	
Men and women	2.0	1.3–3.1

*MSM: men who have sex with men

**aPR: adjusted Prevalence Ratio

***95% CI: 95% confidence interval

When we focused on the first injection episode of those who had ever injected, we observed that 81.9% injected for sex at first injection (FIS) versus 18.1% who injected with other purposes. Some 30.8% of non-FIS also injected drugs for sex later on. FIS participants became injection initiates when they were more than 7 years older than non-FIS (31.1 vs 23.9); 83.1% of FIS had injected for the first time in the last 3 years versus only 46.2% in non-FIS, around 70% in both groups did not self-inject but were injected by someone else; methamphetamine was the first drug injected by a third of the participants from both groups, mephedrone was the initiate drug for 62.7% of FIS, while non-FIS used other different drugs, like cocaine, heroin and amphetamine. The drug used for the first injection was significantly different by city: in Madrid mephedrone was the drug of choice for 77.8%, while in Barcelona it was methamphetamine (78.6%) (Table 3).

**Table 3:** Characteristics of drug injection by purpose of first injection among MSM* in Madrid and Barcelona

	First injection for sex		First injection NON for sex		Total		*p* value
	*N* = 59 (81.9%)		*N* = 13 (18.1%)		*N* = 72 (2.1%)		
	*N*	%	*N*	%	*N*	%	
First injection							
Age: Median (1–3 IQR)	31.1	26.0–35.0	23.9	20.0–25.0	29.8	24.0–34.0	0.002**
*Years since first injection*							0.005
≤ 3	49	83.1	6	46.2	55	76.4	
> 3	10	17.0	7	53.9	17	23.6	
*Who performed the injection*							0.888
Self	17	28.8	4	30.8	21	29.2	
Other person	42	71.2	9	69.2	51	70.8	
*Who was the injector*							0.003
Stable partner	0	0.0	1	11.1	1	2.0	
Casual partner	27	64.3	1	11.1	28	54.9	
Friend/acquaintance	15	35.7	7	77.8	22	43.1	
*Drug injected*							< 0.001
Cocaine	0	0.0	2	15.4	2	2.8	
Heroine or other opiods	0	0.0	1	7.7	1	1.4	
Anphetamine (speed)	0	0.0	1	7.7	1	1.4	
MDMA	1	1.7	0	0.0	1	1.4	
Metanphetamine	19	32.2	4	30.8	23	31.9	
Mephedrone	37	62.7	0	0.0	37	51.4	
Ketamine (K, keta, kei)	1	1.7	1	7.7	2	2.8	
Others	1	1.7	4	30.8	5	6.9	
Lifetime injection							
*Number of days with an injection*							0.405
1	19	32.2	7	53.8	26	36.1	
2–4	11	18.6	1	7.7	12	16.7	
5–19	12	20.3	3	23.1	15	20.8	
≥ 20	17	28.8	2	15.4	19	26.4	
*Last injection*							0.188
Last month	28	48.3	3	23.1	31	43.7	
Last 6 months	17	29.3	4	30.8	21	29.6	
Last 12 months	6	10.3	4	30.8	10	14.1	
> 12 months	7	12.1	2	15.4	9	12.7	
*Drugs ever injected*							
Cocaine	2	3.4	4	30.8	6	8.3	< 0.001
Heroine or other opiods	1	1.7	1	7.7	2	2.8	0.234
Anphetamine	0	0.0	2	15.4	2	2.8	0.002
MDMA	2	3.4	1	7.7	3	4.2	0.482
Metanphetamine	27	45.8	4	30.8	31	43.1	0.323
Mephedrone	40	67.8	1	7.7	41	56.9	< 0.001
Ketamine	12	20.3	2	15.4	14	19.4	0.683
Others	3	5.1	4	30.8	7	9.7	0.005
Sharing drug or injection equipment							
*Ever shared*							0.106
No	36	61.0	11	84.6	47	65.3	
Yes	23	39.0	2	15.4	25	34.7	
*Last time shared*							0.061
Last month	13	56.5	0	0.0	13	52.0	
Last 6 months	5	21.7	0	0.0	5	20.0	
More than 6 months	5	21.7	2	100.0	7	28.0	
*With how many people*							0.908
1	2	8.7	0	0.0	2	8.0	
2–4	10	43.5	1	50.0	11	44.0	
≥ 5	11	47.8	1	50.0	12	48.0	
*Proportion with they shared for sex*							0.009
All	19	82.6	0	0.0	19	76.0	
Not all	4	17.4	2	100.0	6	24.0	

*MSM: men who have sex with men

**Student's *T* Test

When lifetime injection was analyzed, it appears that most of the participants in both groups were sporadic injectors since 32.2% of FIS and 53.8% of non-FIS had only injected once and 28.8% FIS and 15.4% non-FIS had done it twenty or more times. Both groups had similar patterns of drug use for the first injection: FIS had used the ChSD, especially mephedrone (67.8%) and practically never had injected any of the other drugs while non-FIS had moderate levels of injection for both groups of drugs (ChSD and non ChSD) except opioids, MDMA and mephedrone.

39.0% of FIS had shared drugs or injecting equipment during their lifetimes, 78% in the last 6 months; while only 15% of the non-FIS had done so and none of them in the last 6 months. The 47.8% non-FIS who had shared had done so with more than five people, and 82.6% had always shared for sex.

## Discussion

### Main results and comparisons with other studies

As far as we know, this is the first study to provide clear empirical evidence that injecting drugs to have sex is being the most common first episode of injection for MSM. Previous studies [17] have documented the use of injection as an administration route for various different drugs consumed for having sex. However, none of these studies asked whether the first injection was for sex or for any other purpose. This study shows that more than four out of five participants, who had injected drugs during their lifetimes, had done so for sex when injecting for the first time. In addition, of those whose first time injecting was not for sex, one third had injected for sex later. It has been well known for several years that self-intravenous injection is a difficult to adopt behavior, as injection generates fear and instinctive rejection, and also requires training [27, 28]. For both reasons, the first few times novices are injected by or at least under the supervision of a more experienced injector. We could say that a prerequisite for the spread of injecting is that there are skilled injectors.

The study shows that the proportion of MSM who had ever injected is small: Only two per cent. However, when assessing its significance, it is necessary to bear in mind that the spread of injection among MSM is relatively recent, and therefore its growth potential may be substantial. In fact, more than four out of five FIS in the study began injecting in the last 3 years. The prevalence found in our study is higher than the 1.5% found in Spain as a whole by the EMIS study [29], despite the fact that the latter included more than 10% of HIV-positives in its sample, who can be expected to have a higher injection prevalence. This prevalence found by EMIS (an Internet-based survey) in Spain was lower than in some countries, such as the UK, the Netherlands, Belgium and France. Likewise, the prevalence found in the present study is lower than that found in a different Internet-based survey in the UK (2.9%) [15] or in a study in five large cities in France using time-location (3.1%) [14], but higher than another Internet-based survey in Ireland (1.6%) [30]. The comparison with the other studies included in a recent review [18] makes little sense as those studies are qualitative, either with a fairly limited sample size or with very different inclusion criteria. Considering that most injectors in Spain are recent initiates, it could be that this difference in prevalence is mainly due to the fact that injection among MSM has spread later in Spain than in other countries, especially the UK. In any case, we should remember the impact that the adoption of this administration route can have on MSM’s health. It is well known that this risky behavior is much more efficient than sexual practices at transmitting HIV, HBV and HCV [20], and, in addition, for pharmacokinetic reasons, injection is one of the strongest correlates for overdose [21, 22] and for the development of dependence [23].

The two strongest correlates with having ever injected drugs were the number of partners participants had recently been penetrated by and having met those partners mainly at private parties. Both variables clearly indicate that injecting occurs in MSM who frequent what are known as chemsex sessions. In addition, injecting was also associated with having injected steroids, since those who do so have probably lost their "fear of the needle" [27, 31, 32]. The association found with having been paid for sex indicates that this behavior possibly occurs in the context of sex work. A key point is that the association with having also had relationships with women is a wake-up call since some MSM could act as a bridge population for the dissemination of this behavior among women, as happened in the injection epidemic of the 80 s and 90 s in the general population, when most the women who injected were introduced to this administration route by their injecting partner [27].

A surprising finding is that the mean age of the first injection for FIS was over 30 and more than a quarter had started at least 35 years old, while that of non-FIS was roughly 24, more similar to trends in the general population when drugs are injected for any purpose [6].

As is common with drug use, there are differences in local patterns. The prevalence in Madrid was higher than in Barcelona, and in Madrid mephedrone was the most injected, both for lifetime and the first injection, while in Barcelona it was methamphetamine. The higher prevalence of injecting in Madrid than in Barcelona is another indicator that injecting among MSM is a phenomenon quite independent of developments in the general population. In fact, in Barcelona injecting heroin or cocaine as a primary route of use is still almost twice as high as in Madrid in people requesting treatment for the use of these drugs [33, 34].

The high prevalence of sharing drugs or injection equipment should be noted: Almost two out of every five FIS had done so, more than double that of non-FIS. It is not possible to compare this finding with that offered by EMIS [29], since that study presents the prevalence of sharing without differentiating whether participants have injected drugs or steroids and does not offer this information by country. The Spanish EMIS report [35] found 14.8% sharing for all injectors (of drugs or steroids). The study in France [14] mentioned above found 21.5%. Comparisons with the other studies in the review we have already discussed [16] do not seem useful for the reasons already outlined. Finally, it is worth noting that among FIS, more than four out of five have only ever injected for sex. It is unknown to what extent the current low percentage of those who have injected for other purposes may increase in the future, once they have crossed the barrier into needle use.

### Limitations

First of all, it should be borne in mind that like almost all studies in this population, this study employed convenience sampling, in this case MSM accessing HIV testing. In order to increase the sample’s heterogeneity and representativeness, a sample of significant size was recruited and two programs with very different client recruitment characteristics were chosen in each city. Still, caution should be exercised in generalizing results even among HIV-negative MSM, because convenience samples tend to sample higher-risk MSM than general population surveys [36, 37]. However, by not including HIV-positive MSM, it is almost certain that some findings are underestimates of the true prevalences should we generalize the results to all MSM, since HIV-positive MSM have a higher prevalence of injecting in all studies [14, 15, 18]. Likewise, due to the low prevalence of injection overall, the comparison between FIS and non-FIS has certain limitations, with the FIS results being more accurate, due to its higher prevalence. It would be very desirable that the findings of this study could be confirmed by other studies carried out in MSM populations recruited by different methods, not limited to large cities and also including HIV-positive individuals.

## Conclusions

To the best of our knowledge, this is the first study showing that among MSM, injecting for sex is being the most common first episode of drug injection, which is also the most efficient behavior for the transmission of HIV, Hepatitis B or C and other blood-borne infections. However, only two out of a hundred had injected, most very occasionally but for the purpose of having sex, and sharing drugs or injecting equipment was frequent in these events. However, in each of the cities, a different drug predominated. It was not explicitly asked about, but the correlates found point to it occurring in the context of chemsex sessions with multiple partners and often when the new injector is paid for sex. Finally, MSM who also have sex with women are more involved, so there is a risk that it will become the bridge population for the spread of injecting to women, and indirectly to non-MSM, as happened in the injecting epidemic at the end of the last century [27].

MSM participating in private parties, especially those who are paid to have sex, should be considered a priority group for prevention and harm reduction policies. To prevent this risky behavior and minimize its harms it will be necessary to design imaginative programs, since there are no harm reduction strategies that have been evaluated or even designed specifically to target MSM who inject drugs in private sex parties and who receive money in exchange for sex. The most approximate ones are the harm reduction guidelines for slamming during sex sessions [38]. Taking into account the interventions that have been suggested by the participants themselves can also be a good starting point [39, 40]. However, these initiatives should take into account for the challenge posed by the likely existence of culture of “counterpublic health.” This culture has been found to be underpinned by forms of “sex-based sociality,” which gives primacy to the priorities and practices of gay and bisexual men, as a recent study in Australia has shown [41].

## Supplementary Information

**Additional file 1**. Equivalence of original questions and variables used in the analysis.

## Data Availability

The datasets used and/or analyzed during the current study are available from the corresponding author on reasonable request.

## Abbreviations

ChSD: Chemsex drugs
CI: Confidence intervals
FIS: First injection was for sex
MSM: Men who have sex with other men
non-FIS: Non first injection was for sex
PRs: Prevalence ratios
STI: Sexually transmitted infection
