# Supplementary material for: Sexualized drug injection among men who have sex with men in Madrid and Barcelona as the first episode of drug injecting
Source: Harm Reduct J. 2021 Aug 6;18:86. doi: 10.1186/s12954-021-00531-2 (PMC8344139; doi:10.1186/s12954-021-00531-2)
Supplement: Supplementary file 1 — Additional file 1. Equivalence of original questions and variables used in the analysis. [file 12954_2021_531_MOESM1_ESM.pdf]

Annex 1: Original variables and categories from table 1, as they were collected in the questionnaire and their corresponding variables and categories as they have been employed in the analysis. (I)

Original form of variables and categories as used in the questionnaire	Variables and categories employed in the analysis
**Recruitment**	
**City of testing**	**City of testing**
Barcelona	Barcelona
Madrid	Madrid
**Kind of testing program**	**Kind of testing program**
Centro Sanitario Sandoval	STI diagnostic center
UITS Drassanes	
Pink Peace program	Comunity program
Agencia de Salud Pública de Barcelona	
**Sociodemographics**	
**Age (years)**	**Age (years)**
Continuous variable	≤ 24
	25-39
	≥ 40
**Country of birth**	**Country of birth**
Open answer	Spain
	Latin-America
	Others
**Years residing in Spain (for those born outside of Spain)**	**Years residing in Spain (for those born outside of Spain)**
Open answer	≤ 1
	2-4
	≥ 5
**Size of city of residence (last 12 months)**	**Size of city of residence (last 12 months)**
Less than 10 thousand	≤ 100.000
More than 10 thousand	
More than 50 thousand	
More than 100 thousand	100.000 - 1 million
More than 500 thousand	
More than a million	> 1 million
**Level of education**	**Level of education**
None or primary education (the level that should be finished at 12 years of age)	Up to upper secondary
Lower secondary or second stage of Basic education: designed to complete Basic education, usually following a more subject-oriented pattern (the level that should be finished at 16 years of age)	
(upper) secondary education: more specialized education typically beginning at age 15 or 16 years and/or the end of compulsory education (the level that should be finished at 18 years of age)	Post secondary
Post-secondary, non-tertiary education: captures programs that straddle the Boundary between upper- and post-secondary education from an International point of view as pre-university courses of short vocational programs (the level that should be finished at 20 years of age)	
Higher education university education: specific vocational training, first and second university degrees, Bachelor, Master degree, Doctorate (PhD)	University
**Employment status (last 12 months)**	**Employment status (last 12 months)**
Employed full-time	Employed
Employed part-time	
Self-employed	
Unemployed (with or without subsidy)	Unemployed
Student	Others
Long-term sick leave/ medically retired	
Retired	
**Economic situation (last 12 months)**	**Economic situation (last 12 months)**
Very comfortable	Comfortable/ It is OK
Comfortable, it is all right	
It is tight, I need to be careful	Tight
I make ends meet with difficulties	Dificult/Very difficult
I am unable to make ends meet without debt.	
**Cohabitation (last 12 months)**	**Cohabitation (last 12 months)**
Alone	Alone
With some people	With some people
**Sexual and risk behavior**	
**You have ever had sex with....**	**Gender of sex partners (ever)**
Only with men	Only men
More often with men, but at least once with a woman	Men & women
About equally often with men and with women	
More often with women, but at least once with a man	
**Age at first sexual intercourse with another men (years)**	**Age at first sexual intercourse with another men (years)**
Continuous variable	< 16
	16-20
	21-24
	≥ 25

Annex 2: Original variables and categories from table 1, as they were collected in the questionnaire and their corresponding variables and categories as they have been employed in the analysis. (II)

**Original form of variables and categories as used in the questionnaire**	**Variables and categories employed in the analysis**

Sexual and risk behavior

**You live sex-life with men…**	**Lives sex-life with men…**
In total secrecy	
Hidden	Not Openly
Discreetly	
Openly	Openly

**Place where you met the largest number of partners**	**Place where the largest number of partners were met**
Discotheques, clubs and gay bars	Discos/clubs/bars
Saunas	Saunas
Apps	Apps/websites
Internet	
Parks, public restrooms, and other places for flirting or "cruising"	Cruising places
A sex party in a private home	Private parties
Sex Clubs	
Dark Rooms, sex shops	Others/no search
Other: specify	

**Place where you met the largest number of partners**	**Place where the largest number of partners were found**
Discotheques, clubs and gay bars	
Saunas	
Apps	
Internet	
Parks, public restrooms, and other places for flirting or "cruising"	Others
Other: specify	
Sex Clubs	
Dark Rooms, sex shops	
A sex party in a private home	Private parties

**Number of men penetrated by (ever)**	**Number of men penetrated by (ever)**
None	None
One	
Less than 5	
Less than 10	1-49
Less than 20	
Less than 50	
Less than 100	
Less than 200	≥ 50
More than 200	

**Number of men penetrated by (last 12 months)**	**Number of men penetrated by (last 12 months)**
None	None
One	
Less than 5	
Less than 10	≤5
Less than 20	
Less than 50	
Less than 100	
Less than 200	>5
More than 200	

**Number of men who have ever paid you for sex / Occurrence of last episode**		**Been paid for sex**
None →		Never
One		
Less than 5	**When was the last time?**	
Less than 10	In the last month	
Less than 20 →	In the last 6 months →	Last 12 months
Less than 50	In the last 12 months	
Less than 100	In the last 5 years	
More than 100	More than 5 years ago →	> 12 months ago

**Number of men you have ever paid for sex / Occurrence of last episode**		**Paid for sex**
None →		Never
One		
Less than 5	**When was the last time?**	
Less than 10	In the last month	
Less than 20 →	In the last 6 months →	Last 12 months
Less than 50	In the last 12 months	
Less than 100	In the last 5 years	
More than 100	More than 5 years ago →	> 12 months ago

**Ever injected steroids**	**Ever injected steroids**
No	No
Yes	Yes

Annex 3: Original variables and categories from table 1, as they were collected in the questionnaire and their corresponding variables and categories as they have been employed in the analysis. (III)

Original form of variables and categories as used in the questionnaire		Variables and categories employed in the analysis

History of HIV and other STI testing

Number of previous HIV tests / Occurrence of last HIV testing episode		Time since last HIV test
Never		Never
Once		
2 times — **When was the last time?**		
3 to 5 times — In the last month		Last 12 months
6 to 9 times — In the last 6 months		
10 to 15 times — In the last 12 months		
16 to 20 times — In the last 5 years		> 12 months ago
More than 20 times — More than 5 years ago		

HIV new diagnosis in this consultation		HIV new diagnosis in this consultation
Based on the result of the HIV test		No
		Yes

STI diagnosis (ever)		STI diagnosis (ever)
None		No
Syphilis		Yes
Gonorrhoea		
Chlamydia		
Lymphogranuloma venereum		
Anal or genital warts		
Genital or anal herpes		

Annex 4: Original variables and categories from table 3, as they were collected in the questionnaire and their corresponding variables and categories as they have been employed in the analysis. (I)

Original form of variables and categories as used in the questionnaire	Categories grouped for analysis
**First injection**	
**Age**	**Age**
Continuous variable	median (1-3 IQR)
**Years since first injection**	**Years since first injection**
Difference between current age and age of first injection	≤3
	>3
**Who performed the injection**	**Who performed the injection**
Oneself	Self
Other person	Other person
**Who was the injector**	**Who was the injector**
Stable sexual partner	Stable partner
Casual sexual partner	Casual partner
Brother	Friend/acquaintance
Another relative	
Very close friend	
Dealer	
Casual acquaintance	
Others. Specify:	
**Drug injected**	**Drug injected**
Powdered or crack cocaine (theme, lighthouse, base, basuco, etc.)	Cocaine
Heroin (horse) or other opium-derived drugs (poppy straw, liquid heroin, acetylated opium, fentanyl, methadone or others)	Heroine or other opiods
Anphetamine (speed)	Anphetamine (speed)
MDMA (M, crystal) in its crystalline form or in its powder form	MDMA
Methamphetamine (bathtub, crystal meth, T)	Metanphetamine
Mephedrone (mefe) or other different synthetic stimulants (bath salts, methoxetamine/MXE, methylone/3MMC, methylethcathinone/4MEC, fluoroamphetamine/light ecstasy/4FA)	Mephedrone
Ketamine (K, keta, kei)	Ketamine (K, keta, kei)
Others of which I don't know the name or specify	Others
**Lifetime injection**	
**Number of days with an injection**	**Number of days with an injection**
One	1
Less than 5	2-4
Less than 10	5-19
Less than 20	
Less than 50	≥20
Less than 100	
More than 100	
**Last injection**	**Last injection**
In the last month	Last month
In the last 6 months	Last 6 months
In the last 12 months	Last 12 months
In the last 5 years	>12 months
More than 5 years ago	
**Drugs ever injected**	**Drugs ever injected**
Powdered or crack cocaine (theme, lighthouse, base, basuco, etc.)	Cocaine
Heroin (horse) or other opium-derived drugs (poppy straw, liquid heroin, acetylated opium, fentanyl, methadone or others)	Heroine or other opiods
Anphetamine (speed)	Anphetamine
MDMA (M, crystal) in its crystalline form or in its powder form	MDMA
Methamphetamine (bathtub, crystal meth, T)	Metanphetamine
Mephedrone (mefe) or other different synthetic stimulants (bath salts, methoxetamine/MXE, methylone/3MMC, methylethcathinone/4MEC, fluoroamphetamine/light ecstasy/4FA)	Mephedrone
Ketamine (K, keta, kei)	Ketamine
Others of which I don't know the name or specify	Others

Annex 5: Original variables and categories from table 3, as they were collected in the questionnaire and their corresponding variables and categories as they have been employed in the analysis. (II)		
**Original form of variables and categories as used in the questionnaire**		**Categories grouped for analysis**
**Sharing drug or injection equipment**		
**Ever shared**		**Ever shared**
No		No
Yes		Yes
**Last time shared**		**Last time shared**
In the last month		Last month
In the last 6 months		Last 6 months
In the last 12 months		More than six months
In the last 5 years		
More than 5 years ago		
**With how many people**		**With how many people**
One		1
Less than 5		2-4
Less than 10		≥5
Less than 20		
Less than 50		
More than 50		
**Proportion with whom they shared with the purpose of having sex**		**Proportion with they shared for sex**
All		All
More than half		Not all
Half		
Less than half		
None		
